# Elevated Glycated Hemoglobin Levels Are Associated With Poor Outcome in Acute Ischemic Stroke

**DOI:** 10.3389/fnagi.2021.821336

**Published:** 2022-02-03

**Authors:** Nan Dong, Xiaozhu Shen, Xuan Wu, Xianghong Guo, Qi Fang

**Affiliations:** ^1^Department of Neurology, The First Affiliated Hospital of Soochow University, Suzhou, China; ^2^Department of Neurology, Suzhou Industrial Park Xinghai Hospital, Suzhou, China

**Keywords:** acute anterior circulation ischemic stroke, glycated hemoglobin, outcome, atherosclerosis, predictor

## Abstract

**Objective:**

Admission hyperglycemia is an established risk factor for functional outcome in patients with acute ischemic stroke. However, the association between glycated hemoglobin (HbA1c) and prognosis in patients with acute anterior circulation ischemic stroke (AACIS) remains controversial. This study aimed to explore whether elevated HbA1c levels are associated with functional outcome in AACIS patients.

**Participants and Methods:**

We enrolled patients with AACIS hospitalized in the First Hospital Affiliated to Soochow University from March 2018 to January 2021. Patients were categorized into three groups based on baseline HbA1c: HbA1c ≤ 6.5%, 6.5% < HbA1c ≤ 8.0%, and HbA1c > 8.0%. Ninety-day modified Rankin Scale scores of 0–1 and 0–2 were defined as excellent and favorable functional outcome, respectively. Early neurological improvement was regarded as a reduction in the National Institutes of Health Stroke Scale (NIHSS) score ≥ 4 points compared with that on admission, or an NIHSS score of 0–1 at discharge. The association between HbA1c and clinical outcome in acute ischemic patients was assessed by logistic regression and adjusted for confounding factors. Subgroup analyses by TOAST classification were also conducted.

**Results:**

The study included 326 patients. The proportion with favorable outcome was significantly lower in the HbA1c > 8.0% group than the HbA1c ≤ 6.5% group (30.4 vs. 55.2%; *p* < 0.01). Binary logistic regression analysis demonstrated that increasing HbA1c levels (as a continuous variable) were associated with reduced functional independence (adjusted *OR* = 0.739; 95% CI: 0.605–0.904; *p* = 0.003). In subgroup analyses, higher HbA1c was also associated with favorable outcome in large-artery atherosclerosis (LAA)-type patients (adjusted *OR* = 0.776; 95% CI: 0.614–0.981; *p* = 0.034), but not in LAA group.

**Conclusions:**

HbA1c level was an independent predictor of worse functional outcome in patients with AACIS, particularly in those with LAA. For patients with anterior circulation atherosclerosis, strict adherence to a target HbA1c < 6.5% may be required.

## Introduction

It is well-established that acute hyperglycemia on admission is related to unfavorable outcomes in acute ischemic stroke, regardless of intravenous thrombolysis or mechanical thrombectomy (Kim et al., 2016; Osei et al., 2017; Rinkel et al., 2020). Various mechanisms may be involved in this association, including intracellular acidosis, procoagulant state, endothelial dysfunction, or production of reactive oxygen species induced by hyperglycemia contributing to exacerbation of brain injury and reperfusion injury (Martini and Kent, 2007; Suh et al., 2008). However, it remains controversial whether chronic hyperglycemia has the same effect on clinical outcome in patients with acute anterior circulation ischemic stroke (AACIS, Luitse et al., 2017; Sung et al., 2017; Wang et al., 2019), in particular with large-artery atherosclerosis (LAA). Chronic hyperglycemia, also denoted pre-stroke glycemic control (Kamouchi et al., 2011), is usually assessed by glycated hemoglobin (HbA1c), which is a reliable index to estimate average glucose levels over a 2–3-month period. In June 2021, Santos et al. (2021) reported an independent positive association between HbA1c and subclinical atherosclerosis in both prediabetic and non-prediabetic individuals. A cohort study spanning 8.4 years conducted in Hong Kong indicated that the HbA1c level was associated with elevated cardiovascular disease risk (Wan et al., 2020), suggesting a closer relationship between HbA1c and LAA-type stroke than other subgroups. We performed the present study to investigate whether HbA1c is associated with poor functional outcome in patients with AACIS. In addition, we discuss the potential mechanisms in this context.

## Materials and Methods

### Study Population

The present study was conducted in the Stroke Center of the First Hospital Affiliated to Soochow University. We performed a retrospective and partly perspective analysis of collected data during the period March 2018 to January 2021. Patients were enrolled in this study if they met the following criteria: (1) age ≥ 18 years old; (2) acute anterior circulation ischemic stroke onset within 48 h; and (3) presence of acute ischemic lesions in the anterior circulation, confirmed by imaging methods (magnetic resonance angiography or computed tomography). Exclusion criteria for this study were: (1) intracranial hemorrhage or mass lesion; (2) severe infection or septic shock; (3) liver or renal failure; or (4) incomplete laboratory, clinical or follow-up data. There were totally 464 patients with acute ischemic stroke hospitalized in the First affiliated Hospital of Soochow University from April 2018 to April 2021, we excluded 102 patients with posterior circulation infarction, 2 patients with renal failure, 5 patients with severe infection and 29 patients with incomplete follow-up data. Totally 326 patients were enrolled in the study.

This study was approved by the Ethics Committee of the First Hospital Affiliated to Soochow University (No. 2020272, 2019-057). Written informed consent for participation was not required for this study in accordance with the national legislation and the institutional requirements.

### Data Collection and Assessment

Baseline clinical information of all enrolled patients was collected from the stroke center patient database of the First Hospital Affiliated to Soochow University, including age, sex, systolic blood pressure (SBP), diastolic blood pressure (DBP), glucose, National Institutes of Health Stroke Scale (NIHSS) score, history of hypertension, diabetes, atrial fibrillation, prior stroke or transient ischemic attack, family history, and current smoking habits (cigarettes smoked per day in the past 30 days). Laboratory findings included triglycerides (TG), total cholesterol (TC), low-density lipoprotein cholesterol (LDL-C), high-density lipoprotein cholesterol (HDL-C), HbA1c, and homocysteine. TOAST classification was divided into LAA, cardioembolic (CE), and others. LAA was defined as > 50% stenosis of the vessel lumen in extracranial or intracranial segment of internal carotid artery, M1/M2 segment of middle cerebral artery, or anterior cerebral artery. CT angiography (CTA) data were used to assess regional leptomeningeal collateral score to evaluate the collateral status according to Menon et al. (2011). The functional outcome was assessed by 90-day modified Rankin Scale (mRS) after onset of symptoms, which was determined by a trained operator via face-to-face interview. An excellent outcome was defined as 90-day mRS score of 0–1 and a favorable outcome was defined as a score of 0–2. Early neurological improvement was defined as a reduction in NIHSS score ≥ 4 compared with that on admission, or an NIHSS score of 0–1 at discharge.

### Statistical Analysis

Patients were categorized into three groups based on baseline HbA1c: HbA1c ≤ 6.5%, 6.5% < HbA1c ≤ 8.0%, and HbA1c > 8.0%, with reference to a previous study (Laiteerapong et al., 2019). Statistical analysis was performed using SPSS software (IBM SPSS Statistics for Mac, version 26.0; IBM Corp., Armonk, NY, United States). The Kolmogorov–Smirnov test was used to assess the normality of numerical variables; median and interquartile range were used to describe continuous variables if a non-normal distribution. Differences in baseline data were compared using the Kruskal–Wallis or Mann–Whitney U test for continuous variables, and chi-squared test or Fisher’s exact test for categorical variables. Differences in 90-day mRS score proportions were evaluated by the Pearson’s test. We used Bonferroni correction to control type I error in multiple comparisons, and a *p*-value < 0.05/number (0.0167) of comparisons as a threshold for statistical significance. Binary logistic regression models were used to evaluate the association between HbA1c and outcome variables including excellent outcome, favorable outcome, and early neurological improvement. For multivariate logistic regression analysis, we adjusted any confounding variables with a bivariate *p* < 0.10 including age, sex, baseline SBP, baseline glucose, baseline NIHSS score, history of hypertension, diabetes or atrial fibrillation, TC, and LDL-C. A 2-tailed *p*-value < 0.05 was considered significant.

## Results

### Baseline Characteristics

Between March 2018 and January 2021, a total of 326 patients (63.2% male; median age 68 years; interquartile range 56–75 years) were included in the study. Laboratory tests showed that 231 (70.86%) patients had HbA1c ≤ 6.5%, 38 (11.66%) had 6.5% < HbA1c ≤ 8.0%, and 57 (17.48%) had HbA1c > 8.0%. Baseline clinical and biochemical characteristics, stratified by HbA1c level, are summarized in Table 1. Compared with the high HbA1c group, patients with HbA1c ≤ 6.5% had lower TG levels and higher HDL-c levels. Unsurprisingly, baseline glucose levels were positively correlated with HbA1c. In terms of therapy, patients with lower HbA1c levels were more likely to undergo intravenous thrombolysis and (or) endovascular thrombectomy than those with higher HbA1c. There was no difference in age, sex, baseline SBP, DBP, NIHSS score, other medical history, and TOAST classification among the three groups.

**TABLE 1:** Baseline clinical and biochemical characteristics, according to HbA1c levels.

	HbA1c ≦ 6.5%(*n* = 231)	6.5% < HbA1c ≦ 8.0% (*n* = 38)	HbA1c > 8.0% (*n* = 57)	*P*-value
**Characteristic**				
Age, median (IQR), years	67.5 (56.0, 75.0)	69.0 (61.7, 74.0)	66.5 (53.2, 73.0)	0.684
Male, n (%)	146 (62.9)	23 (60.5)	37 (66.1)	0.851
Baseline SBP, median (IQR), mmHg	152.0 (134.0, 172.0)	155.00 (133.0, 174.5)	158.5 (143.5, 183.0)	0.118
Baseline DBP, median (IQR), mmHg	87.5 (77.0, 100.0)	89.0 (79.5, 100.0)	87.0 (80.0, 101.0)	0.777
Baseline glucose, median (IQR), mmol/L	6.41 (5.58, 7.50)	8.85 (7.39, 11.79)	11.36(8.30, 14.86)	0.000
Baseline NIHSS, median (IQR)	9.0 (4.0, 14.0)	5.0 (2.0, 14.3)	7.5 (3.0, 14.8)	0.226
**Medical history**				
Hypertension	144 (62.1)	29 (76.3)	41 (71.4)	0.133
Diabetes	16 (6.9)	26 (68.4)	35 (62.5)	0.000
Atrial fibrillation	87 (37.5)	11 (28.9)	15 (26.8)	0.234
Prior stroke or TIA	21 (9.1)	6 (15.8)	9 (16.1)	0.369
Family history of stroke	52 (22.4)	4 (10.5)	12 (21.4)	0.246
Smoking	75 (32.3)	14 (36.8)	21 (37.5)	0.696
**TOAST classification**				0.139
Large artery atherosclerosis	121 (52.2)	23 (60.5)	38 (67.9)	
Cardiac embolism	80 (34.5)	8 (21.1)	13 (23.2)	
Others	31 (13.4)	7 (18.4)	5 (8.9)	
**Biochemical variables**				
TG, median (IQR), mmol/L	1.17 (0.82, 1.57)	1.29 (1.03, 2.16)	1.36 (1.00, 1.75)	0.004
TC, median (IQR), mmol/L	4.30 (3.68, 4.89)	4.43 (3.57, 5.22)	4.27 (3.79, 5.20)	0.601
HDL-C, median (IQR), mmol/L	1.05 (0.87, 1.21)	0.89 (0.82, 1.07)	0.99 (0.80, 1.23)	0.021
LDL-C, median (IQR), mmol/L	2.70 (2.07, 3.18)	2.85 (2.09, 3.33)	2.76 (2.13, 3.40)	0.842
HCY, median (IQR), μmol/L	11.20 (9.20, 14.00)	11.40 (8.70, 19.85)	10.05 (7.60, 12.18)	0.008
**Collateral status**				0.227
rLMC score < 11	32 (13.8)	1 (2.6)	6 (10.7)	
11 ≦ rLMC score ≦ 15	42 (18.1)	5 (13.2)	12 (21.4)	
rLMC score > 15	158 (68.1)	32 (84.2)	38 (67.9)	
**Artery occlusion/stenosis**				0.963
ICA	58 (25.0)	9 (23.7)	13 (18.3)	
MCA	161 (69.4)	26 (68.4)	38 (67.9)	
ACA	13 (5.6)	3 (7.9)	3 (5.4)	
**Therapy**				0.011
IV rt-PA only	128 (55.2)	17 (44.7)	28 (50.0)	
EVT only	31 (13.4)	6 (15.8)	1 (1.8)	
IV rt-PA + EVT	13 (5.6)	3 (7.9)	1 (1.8)	
Standardized treatment	60 (25.9)	12 (31.6)	26 (46.4)	

*ACA, anterior cerebral artery; DBP, diastolic blood pressure; EVT, endovascular thrombectomy; HbA1c, glycated hemoglobin; HCY, homocysteine; HDL-C, high-density lipoprotein cholesterol; ICA, internal carotid artery; IQR, interquartile range; IV, intravenous thrombolysis; rt-PA, alteplase; LDL-C, low-density lipoprotein cholesterol; MCA, middle cerebral artery; rLMC, regional leptomeningeal collateral; SBP, systolic blood pressure; TC, total cholesterol; TG, triglycerides; TIA, transient ischemic attack.*

We also stratified enrolled patients by functional outcome. Patients with favorable outcome tended to be younger, male, have lower baseline SBP, lower NIHSS score, not have a history of diabetes or atrial fibrillation, lower HbA1c level, and tended to have cardioembolic stroke in terms of TOAST classification (all *p* < 0.05; Table 2).

**TABLE 2:** Baseline clinical and biochemical characteristics, stratified for functional outcome.

	Favorable outcome	Poor outcome	*P*-value
**Characteristic**			
Age, median (IQR), years	65 (53.5, 73.0)	69.5 (60.0, 77.0)	0.002
Male, n (%)	114 (55.3)	92 (44.7)	0.025
Baseline SBP, median (IQR), mmHg	151.0 (134.0, 172.0)	156.0 (141.3, 178.8)	0.027
Baseline DBP, median (IQR), mmHg	87.0 (77.0, 99.0)	89.0 (78.0, 100.8)	0.596
Baseline glucose, median (IQR), mmol/L	6.60 (5.63, 8.35)	7.34 (6.13, 9.79)	0.004
Baseline NIHSS, median (IQR)	4.0 (2.0, 8.0)	12.5 (8.0, 16.0)	0.000
**Medical history**			
Hypertension	100 (46.9)	113 (53.1)	0.069
Diabetes	31 (40.3)	46 (59.7)	0.038
Atrial fibrillation	46 (40.7)	67 (59.3)	0.009
Prior stroke or TIA	21 (9.1)	6 (15.8)	0.369
Family history of stroke	17 (47.2)	19 (52.8)	0.560
Smoking	59 (53.6)	51 (46.4)	0.436
**TOAST classification**			0.000
Large artery atherosclerosis	90 (49.5)	92 (50.5)	
Cardiac embolism	41 (40.6)	60 (59.4)	
Others	34 (79.1)	9 (20.9)	
**Biochemical variables**			
TG, median (IQR), mmol/L	1.25 (0.87, 1.75)	1.17 (0.94, 1.53)	0.293
TC, median (IQR), mmol/L	4.18 (3.58, 4.91)	4.36 (3.75, 5.06)	0.093
HDL-C, median (IQR), mmol/L	1.00 (0.84, 1.19)	1.04 (0.88, 1.23)	0.235
LDL-C, median (IQR), mmol/L	2.68 (1.96, 3.24)	2.79 (2.24, 3.32)	0.087
HCY, median (IQR), umol/L	10.90 (8.80, 13.60)	11.15 (8.82, 14.83)	0.742
**HbA1C**			0.004
HbA1C ≦ 6.5%	128 (55.2)	104 (44.8)	
6.5% < HbA1C ≦ 8.0%	20 (52.6)	18 (47.4)	
HbA1C > 8.0%	17 (30.4)	39 (69.6)	

*DBP, diastolic blood pressure; HbA1c, glycated hemoglobin; HCY, homocysteine; HDL-C, high-density lipoprotein cholesterol; IQR, interquartile range; LDL-C, low-density lipoprotein cholesterol; SBP, systolic blood pressure; TC, total cholesterol; TG, triglycerides; TIA, transient ischemic attack.*

### HbA1c and Functional Outcomes

**FIGURE 1:**
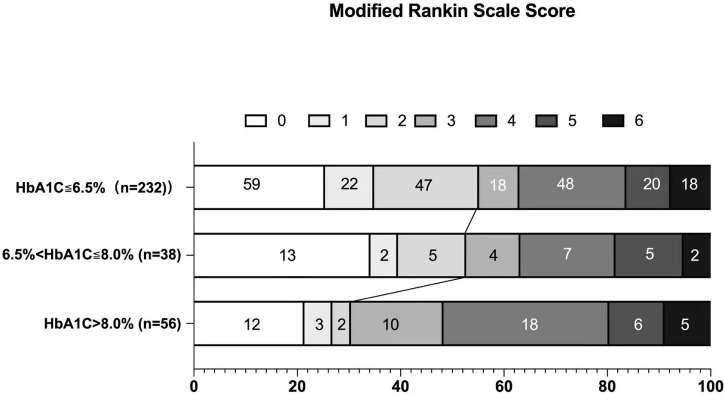
Distribution of scores on the modified Rankin Scale at 90 days.

At 3 months, excellent functional outcome (mRS 0–1) and favorable functional outcome (mRS 0–2) were achieved in 111 (34.04%) and 165 (50.61%) patients, respectively. There was no significant difference regarding excellent functional outcome among the groups (*p* = 0.389, χ^2^ test). In terms of favorable outcome, there were statistically significant differences between the three HbA1c groups: 128 (55.2%) in the HbA1c ≤ 6.5% group, 20 (52.6%) in the 6.5% < HbA1c ≤ 8.0% group, and 17 (30.4%) in the HbA1c > 8.0% group (χ2 = 11.183, *p* = 0.004). Bonferroni correction was further used for pairwise comparison, and we found that favorable outcome occurred more often in the HbA1c ≤ 6.5% group than in the HbA1c > 8.0% group (*p* = 0.004 < 0.0167). No difference was found in the 6.5% < HbA1c ≤ 8.0% group compared with the other groups (Figure 1).

### HbA1c as a Continuous Variable and Outcome

We also used HbA1c as a continuous variable to investigate the association between HbA1c and outcome. As shown in Table 3, overall, lower HbA1c was associated with a shift toward favorable functional outcome with an adjusted odds ratio (OR) of 0.739 (95% CI: 0.605–0.904). Unexpectedly, higher HbA1c was associated with early neurological improvement with OR 1.144 (95% CI, 1.011–1.296), although this was not significant after adjustment for confounding factors in the multivariate logistic regression analysis (*OR* = 1.064; 95% CI: 0.921–1.231), including age, sex, baseline SBP, baseline glucose, baseline NIHSS score, history of hypertension, diabetes or atrial fibrillation, TC, and LDL-C. To determine whether HbA1c levels had an effect on functional outcomes of LAA-type or non-LAA stroke patients, we further analyzed the association between HbA1c and functional outcomes separately for LAA and non-LAA-type infarctions. In the subgroup analysis, multivariate-adjusted ORs for favorable functional outcomes significantly decreased with higher HbA1c in patients with LAA-type infarctions (*p* = 0.034), while no similar pattern was observed in non-LAA-type infarctions. No significant difference was observed in the subtypes between HbA1c and early neurological improvement.

**TABLE 3:** Association between HbA1c levels (as a continuous variable) and functional outcomes in subgroups.

	Univariate logistic regression	Multiple logistic regression*
	OR (95%CI) *P*-value	OR (95%CI) *P*-value
All patients (*n* = 326)		
Excellent outcome (mRS, 0–1)	0.900 (0.786–1.031) 0.117	0.909 (0.750–1.101) 0.329
Favorable outcome (mRS, 0–2)	0.797 (0.697–0.911) 0.001	0.739 (0.605–0.904) 0.003
Early neurological improvement	1.144 (1.011–1.296) 0.033	1.064 (0.921–1.231) 0.399
Patients of LAA (*n* = 182)		
Excellent outcome (mRS, 0–1)	0.898 (0.757–1.065) 0.215	0.960 (0.764–1.205) 0.722
Favorable outcome (mRS, 0–2)	0.787 (0.664–0.934) 0.006	0.776 (0.614–0.981) 0.034
Early neurological improvement	1.171 (0.998–1.374) 0.053	1.094 (0.909–1.318) 0.342
Patients of non-LAA (*n* = 144)		
Excellent outcome (mRS, 0–1)	0.905 (0.718–1.140) 0.397	0.795 (0.529–1.195) 0.271
Favorable outcome (mRS, 0–2)	0.813 (0.650–1.017) 0.070	0.644 (0.402–1.030) 0.067
Early neurological improvement	1.081 (0.880–1.327) 0.460	0.991 (0.748–1.311) 0.947

*Adjusted for age, sex, baseline systolic blood pressure, baseline glucose, baseline National Institutes of Health Stroke Scale score, history of hypertension, diabetes or atrial fibrillation, total cholesterol and low-density lipoprotein.

*HbA1c, glycated hemoglobin; LAA, large-artery atherosclerosis; mRS, modified Rankin Scale.*

## Discussion

This current study indicated that elevated HbA1c was independently associated with unfavorable functional outcomes in patients with AACIS. The occurrence of a favorable outcome significantly decreased in patients with HbA1c > 8.0% as a categorical variable, compared with those with HbA1c ≤ 6.5%. Further analysis using HbA1c as a continuous variable showed that this association differed according to the ischemic-stroke subtype. The association between HbA1c and poor functional outcome was maintained even after adjustment for confounding factors in patients with the LAA subtype, while no similar pattern was observed in non-LAA-type infarctions. No significant difference was observed in the subtypes between HbA1c and early neurological improvement.

In accordance with previous studies, the present study showed that the HbA1c level was associated with an increased risk of unfavorable functional outcome after ischemic stroke (Kamouchi et al., 2011; Luitse et al., 2017; Diprose et al., 2020). However, we found no relationship between HbA1c and early neurological improvement, which is in contrast to previous reports where elevated HbA1c was negatively associated with early neurological improvement (Choi et al., 2018; Diprose et al., 2020). This may be because we did not select patients on the basis of a specific treatment method, and a study population with therapy homogeneity is required to clarify this observation.

Notably, in the subgroup analysis, we found that HbA1c was associated with poor clinical prognosis in patients with LAA-type infarctions, but not in patients with non-LAA-type infarctions. According to European Society of Cardiology guidelines (Piepoli et al., 2016; Cosentino et al., 2020), individuals with HbA1c 5.7–6.4%, even without other known cardiovascular disease risks, warrant risk scoring for primary prevention of cardiovascular disease, reminding us to pay attention to glucose levels even in the pre-diabetes stage. A recent large cohort study enrolled asymptomatic individuals without diabetes and with a low or moderate cardiovascular risk. It identified a positive association between HbA1c and the prevalence and multi-territorial extent of subclinical atherosclerosis (Rossello et al., 2021). For this reason, we speculated that HbA1c may have a greater impact on the prognosis of atherosclerotic stroke compared with other subtypes, and our results confirm this hypothesis. A previous study failed to find evidence of heterogeneity between outcomes when grouping according to CE and non-CE subgroups, in which the LAA-type accounted for only 20% of the CE group. In our study, CE stroke accounted for the majority of non-LAA-type stroke. Nevertheless, since the *P*-value for favorable outcome for non-LAA patients was non-significant in our study, the multivariable-adjusted OR was enough low (0.664). We speculate that the non-significant association in non-LAA subgroup may be due to a type II error for lack of statistical power, thus, these results should be interpreted with caution; particularly in the subgroup analysis.

Early in 2003, Baird et al. (2003) indicated that persistent hyperglycemia is an independent determinant of infarct expansion, and is associated with poor functional outcome. There are a number of possible underlying mechanisms involved in this association. First, chronic hyperglycemia may cause endothelial injury by the deposition of terminal glycosylation products in the vessel wall (Xu and Zou, 2009). In fact, it has been reported that patients with diabetes show vascular injury mainly in the endothelium, while not on the vascular wall (Zhu et al., 2021). Furthermore, Yoda et al. (2014) demonstrated that poor glycemic control could cause morning blood pressure surges, which may accelerate vascular injury synergistically by increasing inflammation in atherosclerotic lesions (Marfella et al., 2007). Second, hyperglycemia can probably lead to neurotoxicity and procoagulant states that can further compromise blood supply in the ischemic areas after stroke. Garg et al. (2006) found that insulin infusion can reverse the injurious effect, not only by lowering blood glucose, but also by resistance of oxidation and inflammation together with improving nitric oxide (NO) production. In addition to the classical mechanisms mentioned above, a recent study showed that hyperglycemia can induce trained immunity and promote macrophage polarization from anti-inflammatory types (M2 type) to pro-inflammatory subtypes (M1 type), which may aggravate the progression of atherosclerosis (Garg et al., 2006; Edgar et al., 2021). This so-called “hyperglycemic memory” has been recently further confirmed (Thiem et al., 2021).

Obviously, in this study, only part of the patients with HbA1c > 6.5% were previously diagnosed with diabetes, which suggests that diabetes was under diagnosed in the real world, therefore, regular blood glucose testing in population with high risk of stroke is supposed to be necessary, even in patients without previously diagnosed diabetes. Also, a larger sample size clinical trial enrolled with patients diagnosed with diabetes mellitus are warranted to confirm this conclusion.

There are some limitations to our study that should be recognized. First, there may be selection bias in this single-center study. Patients with HbA1c ≤ 6.5% accounted for the majority of the cohort, and the small sample size limits the analysis. Furthermore, we did not evaluate secondary outcomes such as mortality or hemorrhage, so this should be addressed in future studies. Since we did not stratify the analyses according to medication/therapy used in the patients, larger studies are needed in the future.

## Conclusion

In brief, our findings indicate that higher HbA1c is independently associated with poor functional outcome in patients with AACIS, particularly in patients with LAA. Thus, it seems to be reasonable that in patients with anterior circulation atherosclerosis, strict adherence to a target HbA1c < 6.5% should be suggested.

## Data Availability Statement

The original contributions presented in the study are included in the article/supplementary material, further inquiries can be directed to the corresponding authors.

## Ethics Statement

The studies involving human participants were reviewed and approved by the Ethics Committee of the First Hospital Affiliated to Soochow University (No. 2020272). Written informed consent for participation was not required for this study in accordance with the national legislation and the institutional requirements.

## Author Contributions

QF and XG conceived and designed the research. ND analyzed the data and drafted the manuscript. ND, XS, XW, and QF collected the data and performed the research. All authors reviewed and edited the manuscript and approved the final version of the manuscript.

## Conflict of Interest

The authors declare that the research was conducted in the absence of any commercial or financial relationships that could be construed as a potential conflict of interest.

## Publisher’s Note

All claims expressed in this article are solely those of the authors and do not necessarily represent those of their affiliated organizations, or those of the publisher, the editors and the reviewers. Any product that may be evaluated in this article, or claim that may be made by its manufacturer, is not guaranteed or endorsed by the publisher.
